# Effect of loading rate and source of energy on the drying parameters of the basil during drying

**DOI:** 10.1038/s41598-023-41697-y

**Published:** 2023-09-08

**Authors:** El-Sayed G. Khater, Adel H. Bahnasawy, Wael Abbas, Osama M. Morsy

**Affiliations:** 1 Agricultural and Biosystems Engineering Department, Faculty of Agriculture, Benha University, Moshtohor, P.O. Box 13736, Toukh, Kalubia Egypt; 2 Basic and Applied Science Department, College of Engineering and Technology, Arab Academy for Science and Technology and Maritime Transport (AASTMT), P.O. Box 2033, Cairo, Egypt

**Keywords:** Ecology, Environmental sciences, Engineering

## Abstract

The basil leaves were dried different sources of energy at different loading rates. Using hybrid solar drying compared to the conventional source of energy such as fossil and propane. Drying parameters were studied. Also, product quality was assessed under study treatments. The results indicated that the higher accumulated weight loss of basil leaves (75.56%) were obtained at 25 kg m^−2^ loading rate and solar drying system. The highest rate of the decrease in moisture content of basil leaves was happened at the 45 kg m^−2^ loading rates. Meanwhile, the lowest rate of the decrease in moisture content of basil leaves was found at 15 kg m^−2^ loading rates. The highest drying rate of basil leaves (219.54 g_water_ kg^−1^ h^−1^) was obtained at the loading rate of 15 kg m^−2^. The highest values of total chlorophyll and color of basil leaves 745.9 and 36.35 were found for solar dryer. The lowest values of total chlorophyll and color of basil leaves 703.5 and 31.66 were found for diesel dryer. The drying efficiency ranged from 33.98 to 40.33% for all batch loads. The highest essential oil yield obtained for solar dryer, the lowest essential oil yield occurred for diesel dryer. The highest value of volatile compounds with found for solar dryer. The total costs for basil drying were 19.73, 26.70 and 23.93 LE h^−1^ for solar, diesel and propane dryers, respectively. Also, the total costs of basil drying were 8.77, 13.15 and 12.27 LE kg^−1^ dried for solar, diesel and propane dryers, respectively.

## Introduction

Herbal plants are cultivated in Egypt for local consumption and export as well and it is considered as one of the most important aromatic plants in Egypt. Its area is about 2121.25 ha, producing about 9031.82 ton/year from green plants and 135.46 ton essential oil^1^.

Basil (*Ocimum basilicum* L.) belongs to the family Lamiaceae is an annual, herbaceous, white to purple flowering plant, 20–60 cm tall, that originated in Iran and India^2,3^ and is considered a main source of essential oil in some countries. Sweet basil is used for many years to flavour foods, as an ingredient of dental and oral health care products and in fragrances^4^. Sweet basil leaves are used to improve the taste of foods like pasta, salads, pizza, meat and often products^5–7^.

Drying is the best way of prolonging the shelf-life of products by inhibiting the microorganisms growth, also it preserves aroma, appearance and nutritional properties. The oxidation reactions which results in drying process of basil may cause reduction in basil volatile content or changing in its nature^8–10^.

The intensity of solar radiation, the temperature, humidity and velocity of the air are continuously changing during sun drying. Therefore, the dried product is affected by drying conditions, where moisture content and drying rate vary based on drying air temperature and fresh product properties. So that, it is important to take into consideration of these factors or conditions^11^.

Solar dryers are environmentally friendly and free of pollution and its designs depend on capability, desire and availability of materials resources. The drying of medicinal and aromatic plants is used extensively and effectively. Investigations in this area provide the optimum conditions for this type of drying all over the world. In Africa, there are many solar drying designs that can be classified as direct, indirect and hybrid solar dryers^12^. Using these dryers optimally is the main challenges for drying different kinds of products.

Hossain et al.^13^ reported that using hybrid solar drying is a good way to dry plants continuously which assure no spoilage or infestation through the night time or off sunshine hours. The use of solar radiation as the only source of energy is recommended for small-scale dryers but the risk of spoilage of big quantities of crops due to bad weather is exist^14,15^. If large scale solar dryers are used for commercial purposes, it is strongly recommended to use artificial dryers or hybrid solar dryers. A huge advantage of solar dryers is the fact that different types of fruits and vegetables can be dried. The quality of products dried in this way is excellent, due to the fact that the food is not in direct sunlight (cabinet or in-house dryer), and due to a shorter drying process-up to a 0.3 of the time in as compared to normal sun drying^16^**.**

The process of solar drying of food continuously by supplementing heat during the night time or low radiation times is very important to avoid the spoilage by microbial the solar dryers performance by adding heat during low radiation times could increase the drying efficiency and keep the product quality. To keep the required drying temperature, storage and supplementary heat sources are assessed^17–19^.

Food drying is one of the most extensive processes in energy usage which consumes from 12 to 25% of the total energy of the industrial processing. Conventional energy caused many problems such as pollution and greenhouse gas emission, so, seeking alternative source of energy such as solar energy will reduce the pollution, keep the quality and reduce the cost of drying process. Also, the loading rater of drying trays is one of the main affecting the drying efficiency and product quality, therefore, the main aim of this study is to study the possibility of using solar energy in drying and compare it with conventional types of energy under different loading rates and investigate there effect on the drying parameters of basil leaves.

## Materials and methods

The experiment was conducted out at the Agri. and Bio-Systems Eng. Dept., Fac. of Agri. Moshtohor, Benha Univ., Egypt (latitude 30° 21′ N and 31° 13′ E), during the period of June and July, 2020. The ambient air temperature ranged from 29.2 to 35.4 °C, the relative humidity ranged from 39 to 71% and solar radiation ranged from 343.6 to 965.7 kJ m^−2^ day^−1^.

### Materials

**Figure 1:**
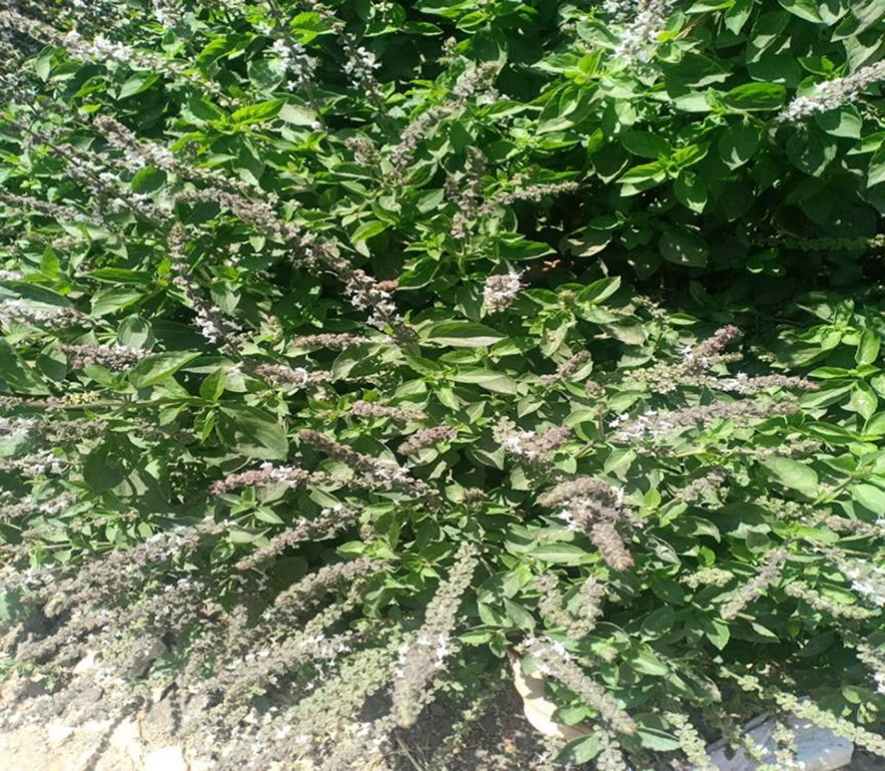
The basil experimental field.

Fresh basil plants that used in the experiment were obtained from the Experimental Station of the Agriculture College, Benha University which is the same place that conducted the experiment at it. Basil (*Ocimum basilicum* L.) belongs to the family Lamiaceae is an annual, herbaceous, white to purple flowering plant, 20–60 cm tall, that originated in Iran and India and has become a major essential oil cultured commercially in some countries. Sweet basil is a popular culinary herb and its essential oil has been used for many years to flavour foods, as an ingredient of dental and oral health care products and in fragrances (Fig. 1).

#### Drying systems description

**Figure 2:**
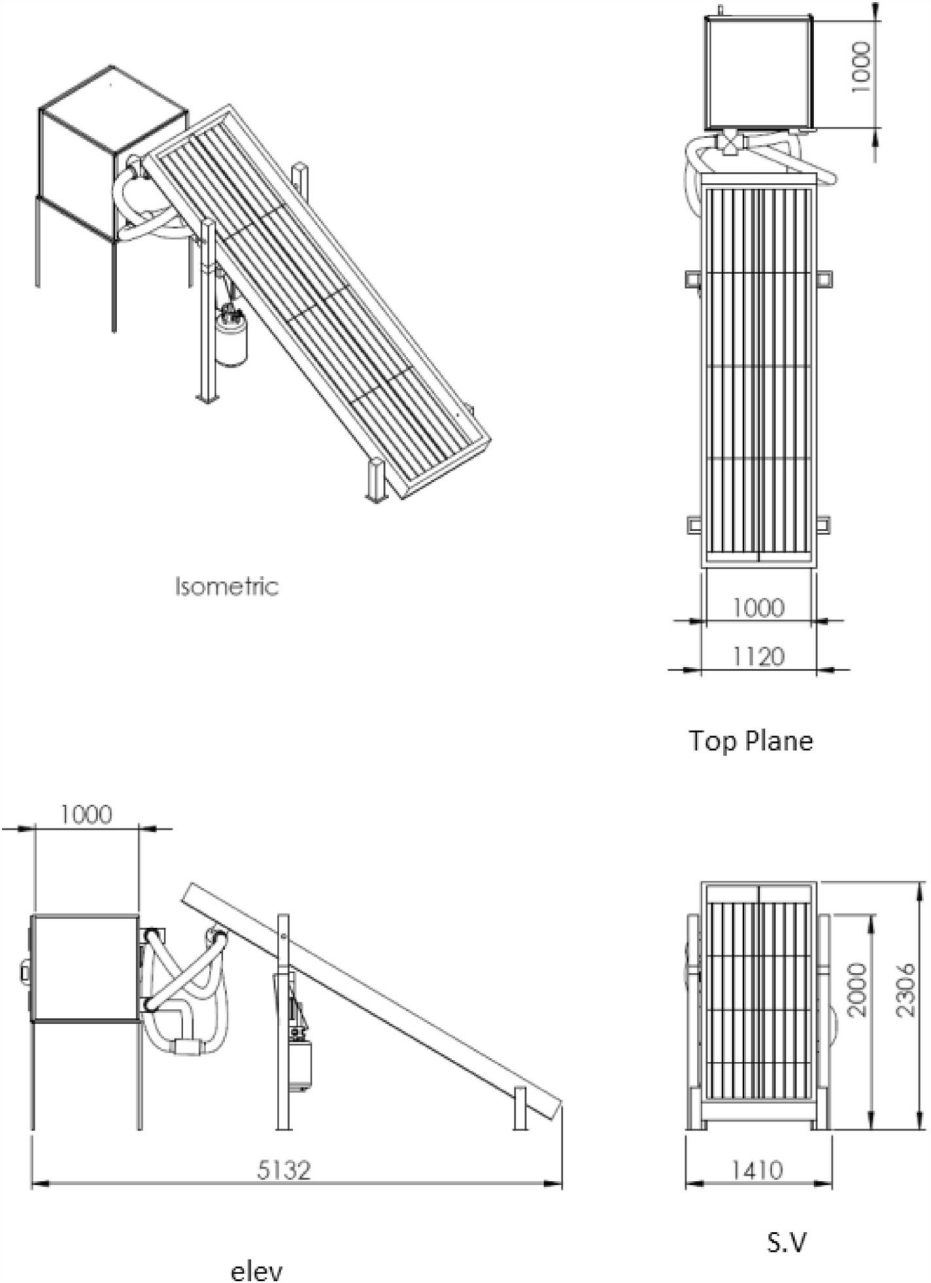
Engineering drawing of the hybrid solar dryer (dimension in mm, Scale 1:100).

A hybrid type of solar drying system was used in this study. This system used three types of energy sources, namely, solar collector, collectors plus supplementary diesel fuel and supplementary propane gas fuel. Figure 2 shows the solar drying system description. The system consists of solar collector, drying chamber, trays, blower and burner.

The features of these components could be described as follows:

- The solar collector

**Figure 3:**
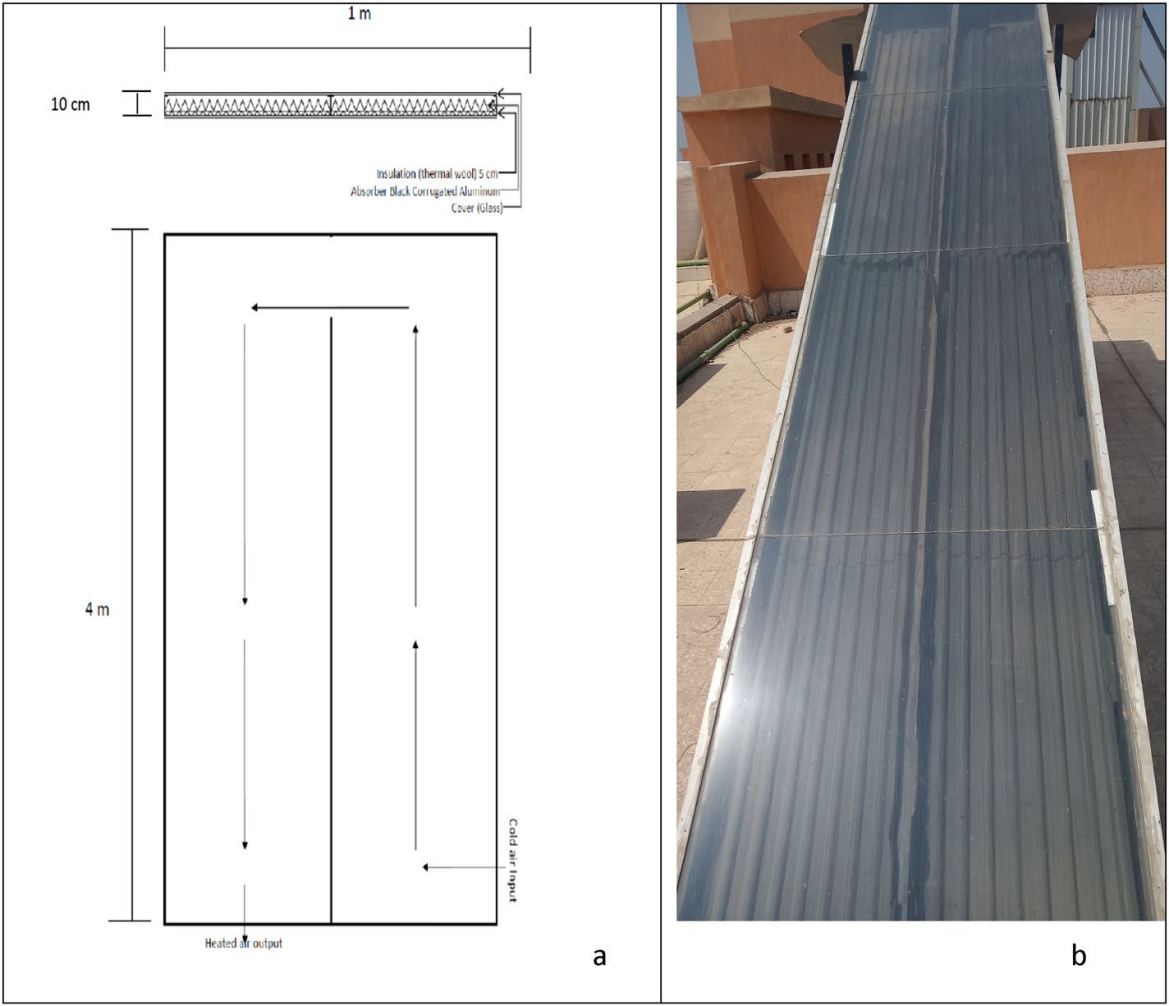
Solar collector. (**a**) Geometric view (**b**) Top view.

The solar collector consists of three major components, namely: The glass cover has dimensions of 4.0 m long, 1.0 m width and 5.5 mm thickness. The cover is fixed on a wooden frame with a thickness of 10 cm. It is divided into two lanes, 50 cm wide each. The absorber plate is made from corrugated black aluminum plate. The insulation is a thermal wool with a 5.0 cm thickness as shown in Fig. 3.

- The drying chamber

**Figure 4:**
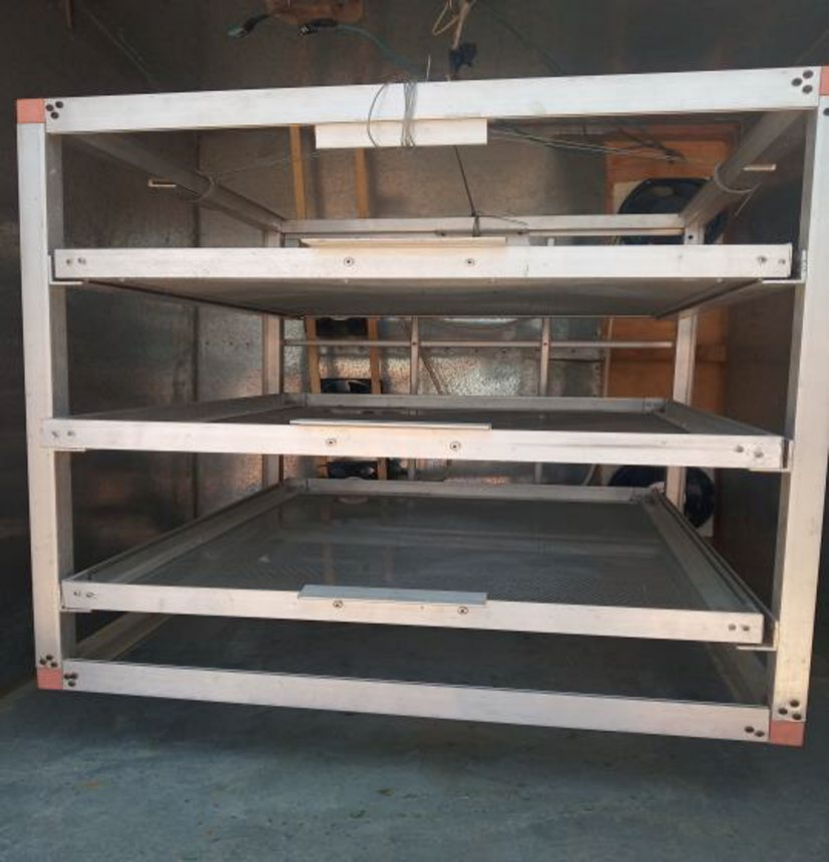
The drying chamber.

The drying chamber has a length of 1.0 m, width of 0.75 m and height of 1.0 m. It is made of galvanized steel (5 mm thickness). The inner surface of drying chamber is covered an insulated materials to reduce heat loss from the walls as shown in Fig. 4.

- The drying trays

The trays are made of stainless steel and have a length of 0.90 m, width of 0.65 m and height of 0.25 m. They have perforated bottom which allows heated air to pass through products.

- Air blower

Two air blowers were used to force and re-circulate the drying air to the drying chamber (Model C.C.P. Parma—Flow Rate 6.6 m^3^ h^−1^—RPM 2800—Power 150 W, 220 V 50 Hz, Italy).

- The burner

The dryer uses a chamber burner system in which heat is being produced. The burner incorporates switches with a sparking mechanism that ignites the fuel when it is fed from the fuel bottle. The system has two types of fuel, propane gas fuel bottle and tank of providing diesel fuel.

- Control units

The control unit is used to control air drying temperature and air recirculation rate. It is consists of data logger and two control units. The data logger. First control unit is used to control of gates and directions of airflow and second is used to control the fuel feeding rate to the burner.

- Air flow directions control gates

The system contains seven gates to control the direct of air flow as follows:

1. Gate (1) from outlet of dryer chamber to inlet of solar collector.
2. Gate (2) from outlet of solar collector to inlet of dryer cabinet.
3. Gate (3) from outlet of dryer cabinet to inlet of gas burner.
4. Gate (4) from out area (fresh air) to inlet of solar collector.
5. Gate (5) from outlet of dryer cabinet to out area.
6. Gate (6) from solar collector to inlet of gas burner.
7. Gate (7) from outlet of burner to inlet of dryer cabinet.

The gates were controlled according to Table 1.

**Table 1:** Control of gates.

T_1_	> T_1 adj._ then	Open gate (2) and (1). **(Sun)**	
	< T_1 adj._ then	Open gate (6), (7) and (3). **(Gas burner)**	
T_2_	> T_2 adj._ then	Sun	Open gate (5), (4) and (2)
		Gas burner	Open gate (5), (4) and (2)
	< T_2 adj._ then	Sun	Open gate (1) and (2)
		Gas burner	Open gate (3), (7) and (6)
H_2_	> H_2 adj._ then	Sun	Open gate (5), (4), (2) and (1)
		Gas burner	Open gate (5), (4), (6), (7) and (3)
	< H_2 adj._ then	Sun	Open gate (1) and (2)
		Gas burner	Open gate (7) and (3)

T_1_ is temperature in outlet solar collector. T_2_ is temperature in dryer cabinet. H_2_ is relative humidity in dryer cabinet.

### Methods

**Figure 5:**
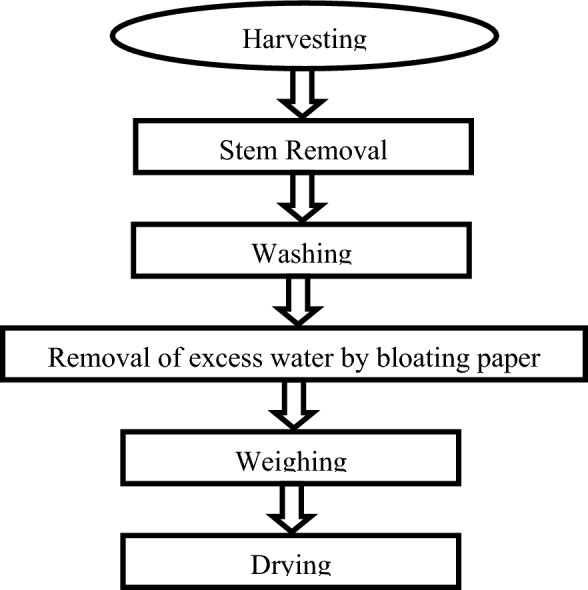
Flow chart of basil processing.

Basil was inspected, sorted and cleaned by removing undesired stems and any waste materials as shown in the process flow chart (Fig. 5). All methods were carried out in accordance to the guidelines and regulations of Benha University. The optimum conditions of basil drying are 60 °C and 10% relative humidity.

#### Experimental procedures

Experiments were divided into two parts: First part was devoted to determine the optimum loading rate of basil leaves to ensure high drying quality and performance. This could be achieved by four batch loads (15, 25, 35 and 45 kg m^−2^). Second part was devoted to determine the energy required for basil drying from different sources of heat. This could be achieved by using three sources of energy (solar energy, propane and diesel fuels).

Weight loss was determined by electric digital balance (Model HG—5000—Range 0–5000 g ± 0.01 g, Japan) hourly for solar and hybrid solar drying methods. Temperature and relative humidity were recorded by using a HOBO Data Logger (Model HOBO U12 Temp/RH/Light—Range − 20 to 70 °C and 5 to 95% RH, USA) every hour. Fuel consumption will be recorded for both diesel and propane fuel sources by weighing the quantity of fuel consumed during drying.

#### Performance analysis

Performance analysis was determined by calculating the moisture content changes, drying rate and drying efficiency as well as energy consumption.

- Moisture content

Moisture content (MC) of the fresh and dried basil leaves was obtained by using oven drying methods according to^20^, where samples were weighed fresh and weighed dried when kept at 105 °C for 24 h or constant weight using the following equation:

1 $$ M{\text{C}} = \frac{{{\text{M}}_{{{\text{wet}}}} - M_{dry} }}{{{\text{M}}_{{{\text{dry}}}} }} \times {100,} $$

where: MC is the moisture content, % d.b. M_wet_ is the wet mass of samples, g. M_dry_ is the dry mass of samples, g.

- Drying rate

The drying rate (DR) of basil was determined as follows according to^14^:

2 $$ DR = \frac{{{\text{M}}_{{{\text{t}} + {\text{dt}}}} - M_{t} }}{{{\text{dt}}}} \, $$

where DR is the drying rate, (kg_water_/kg_dry base_.h). M_t_ is the moisture content at any time t, % d.b. M_t+dt_ is the moisture content at t + dt, % d.b.

#### The quality of dried product

- Total chlorophyll content

Spectrophotometric method according to^21^ was used to determine the chlorophyll (a) and (b) in fresh and dried peppermint leaves as follow: about 500 mg of fresh and 100 mg of dried samples were collected from all the treatments. Samples with 500 mg weight of fresh basil and 100 g of dried basil were taken from each treatment.

Acetone (80%) was added to sample at the rate of 3 ml and then ground using a pestle to extract chlorophyll pigments. Centrifugal tube was used to clarify the samples for 3 min. The clarified sample was dried into volumetric flask and the volume was made up known volume with acetone 80%. The absorbance values were determined at 663 to 644 nm, against a blank of acetone 80%. Chlorophyll (a), chlorophyll (b) and total chlorophyll content were determineded using Eqs. (3, 4 and 5) and expressed in mg/g dry weight.

3 $$chl, a= \left[(12.25{A}_{663}-2.79{A}_{644}\times \frac{V}{W\times 1000\times a})\times \frac{100}{100-M}\right]$$

4 $$chl,b= \left[(21.50{A}_{644}-5.10{A}_{663}\times \frac{V}{W\times 1000\times a})\times \frac{100}{100-M}\right]$$

5 $$ {\text{Total}}\,{\text{chlorophyll }} = {\text{ chl}}.\,{\text{a }} + {\text{ chl}}.\,{\text{b}} $$

where: A is absorbance at specific wavelengths, (644 and 663 nm). V is final volume of chlorophyll extract, (ml). W is weight of the sample, (g). a is path length of light, (1 cm).

- Color analysis

Minolta (CR-400) chromameter (Japan) was used to determine the colors of both fresh and dried spearmint plants. The results in numeric values for three chromatic scales (L*, a*, b*), where L* is the brightness ranging from no reflection for black (L* = 0) to perfect diffuse reflection for white (L* = 100). The value "a*" is the redness ranging from negative values for green to positive values for red. The value "b*” is the yellowness ranging from negative values for blue and positive values for yellow. The color at the grid origin (a* = 0 and b* = 0) is achromatic (gray) as described by^22^**.** A special white plate was used to calibrate the chromameter. Three replicates were taken. The color difference was estimated by Eq. (6):

6 $$\Delta E= \sqrt{{({L}_{i}^{*}-{L}_{0}^{*})}^{2}+({a}_{i}^{*}}-{{a}_{0}^{*})}^{2}+({b}_{i}^{*}-{{b}_{0}^{*})}^{2}$$

where ΔE is the color difference, L_i_* and L_0_* are brightness values for dried and fresh plants, respectively, a_i_* and a_0_* are greenness-redness values for dried and fresh plants, respectively, and b_i_* and b_0_* are blueness-yellowuess values for driod and fresh plants, respectively.

- Extraction of essential oil

Steam distillation method was used to determine the essential oil from fresh and dried spearmint leaves for 3 h as described by^23^**.** Spearmint essential oil of fresh and dried sample. The essential oils were dried oven anhydrous sodium sulphate and stored at -18 °C, till analysis.

- Drying efficiency

The drying efficiency was calculated by Eq. (7) according to^24^:

7 $$ \eta = \frac{{Net\,{\text{energy}}}}{{{\text{Total}}\,{\text{consumption}}\,{\text{energy }}}} $$

8 $$ Net\,{\text{energy}} = m_{w} {\text{I}}_{{\text{v}}} $$

9 $$ {\text{ m}}_{{\text{w}}} = m_{a} \left( {W_{o} - W_{a} } \right) $$

where m_w_ is the evaporation rate, kg s^−1^. I_v_ is the evaporative latent heat, kJ kg^−1^. m_a_ is the mass airflow rate in the dryer, kg s^−1^. W_o_ is the dryer outlet absolute humidity when sensible heat is produced, kg kg^−1^. W_a_ is the inlet absolute humidity in drying chamber, kg kg^−1^. Total consumption rate was calculated for each source of energy as (for diesel 44,000 kJ kg^−1^, for propane 46,350 kJ kg^−1^).

- Chromatographic analyses

DsChrom 6200 Gas Chromatograph equipped with a flame ionization detector for separation of volatile oil constituents was used to determine the volatile oil. The chromatograph apparatus was fitted with capillary column DB-WAX 122-7032 Polysillphenylene-siloxane 30 m × 0.25 mm ID × 0.25 µm film. Temperature program ramp increase with a rate of 13 °C/min and 330 ml/min for air. Detector and injector temperature were 280 °C and 250 °C, respectively. The obtained chromatogram and report of GC analysis for each sample were analyzed to calculate the percentage of main components of volatile oil^7^.

### Cost calculation for the drying basil

The costs were calculated according to^25^. Table 2 shows inputs of drying cost components for three dryers operate by different energy sources (solar, diesel and propane energies) for basil drying.

**Table 2:** Inputs of costs calculations.

Item	Solar dryer	Diesel dryer	Propane dryer
Raw materials (kg)	25	25	25
Fuel consumption (kg)	–	5.1	2.1
Fuel price (L.E)	–	6.7	8
Dried product (kg)	2.25	2.03	1.95
Drying ratio	11.1	12.32	12.82
Experimental period (h)	42	4.6	4
Drying temperature (°C)	39–47	60	60

Density of diesel = 0.85 kg L^−1^.

### Statistical analysis

The data were subjected to analysis using statistical package SPSS version 21 in which one way ANOVA and Duncan Multiple Range Test (DMRT) were performed at significance level of (p < 0.05) at 95% confidence limit to know the significant differences between the treatment means for different parameters.

## Results and discussion

### Weight loss

**Figure 6:**
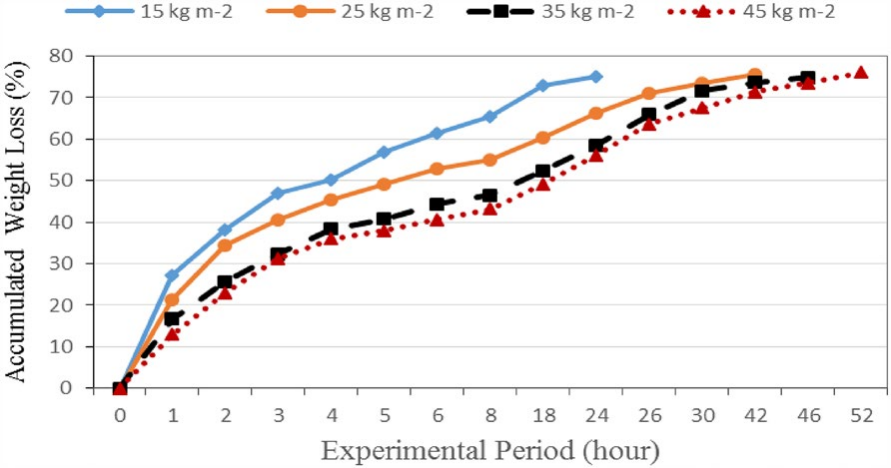
The accumulated weight loss of basil leaves at different loading rates during experimental period.

Figure 6 shows the accumulated weight loss of basil leaves that dried at different loading rates (15, 25, 35 and 45 kg m^−2^). The results indicate that the accumulated weight loss of basil leaves increases with increasing drying period. It could be seen that the accumulated weight loss of basil leaves significantly increased from 27.29 to 75.03%, when the drying period increased from 1 to 24 h, respectively, at 15 kg m^−2^ loading rate. At 25 kg m^−2^ loading rate, the accumulated weight loss of basil leaves significantly increased from 21.45 to 75.56%, when the drying period increased from 1 to 42 h, respectively. At 35 kg m^−2^ loading rate, the accumulated weight loss of basil leaves significantly increased from 16.74 to 74.91%, when the drying period increased from 1 to 46 h, respectively and at 25 kg m^−2^ loading rate, the accumulated weight loss of basil leaves significantly increased from 12.92 to 76.12%, when the drying period increased from 1 to 52 h, respectively. These results agreed with those obtained by^26^.

The results showed that increasing the batch load in a drying chamber, the temperature was decreased. This is due to increasing the evaporation from the plant with increasing load leading to decreasing the dryer temperature and bulk temperature of basil leaves. Drying batch loading rate 15 kg m^−2^ basil leaves consumed 24 h for drying, while 25, 35 and 45 kg m^−2^ consumed 42, 46 and 52 h, respectively, for drying. These results agreed with those obtained by^27^. Also, this is consistent with^28^ who found that the required drying time increases with increasing surface load and small size herbs without stem need low drying time.

The results indicate that the accumulated weight loss of basil leaves decreases with increasing loading rate of basil. It could be seen that, when the loading rate increased from 15 to 45 kg m^−2^, the accumulated weight loss of basil leaves significantly decreased from 27.29 to 12.92%, respectively, after1 hour operated for solar dryer. Also, it significantly decreased from 75.03 to 56.11%, when the loading rate increased from 15 to 45 kg m^−2^, respectively, after 24 h operated for solar dryer.

The results also indicate that the shorter drying period (24 h) was occurred at the 15 kg m^−2^ loading rate. Meanwhile, the longer drying period (52 h) was occurred at the 45 kg m^−2^ loading rate. The results show the highest rate of weight loss was occurred at the first hour. It could be seen that the weight losses were 27.29, 21.45, 16.74 and 12.92% at 15, 25, 35 and 45 kg m^−2^ loading rate, respectively.

Multiple regression analysis was carried out to obtain a relationship between the accumulated weight loss as dependent variable and different both of loading rate and experimental period as independent variables. The best fit for this relationship is presented in the following equation:-

10 $$ WL = 51.60 - 0.62LR + 1.49T\quad {\text{R}}^{{2}} = 0.95, $$

where WL is the accumulated weight loss, %. LR is the loading rate of basil, kg m^−2^. T is the experimental period, h.

This equation could be applied in the range of 15–45 kg m^−2^ loading rate and from 1 to 24 h of experimental period.

**Figure 7:**
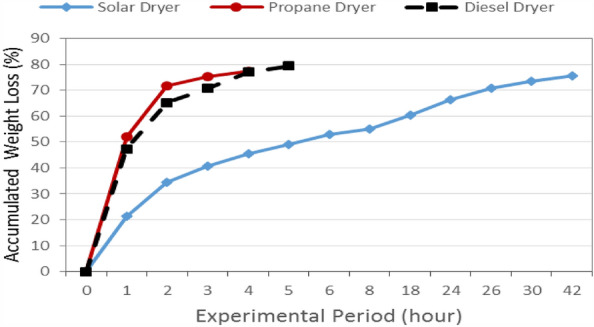
The accumulated weight loss of basil leaves that dried under different energy sources.

Figure 7 shows the accumulated weight loss of basil leaves that dried under different energy sources (solar, propane and diesel energies). The results indicate that the accumulated weight loss of basil leaves increases with increasing drying period. It could be seen that the accumulated weight loss of basil leaves significantly increased from 21.45 to 75.56%, when the drying period increased from 1 to 42 h, respectively, for solar dryer. For diesel dryer, the accumulated weight loss of basil leaves significantly increased from 52.19 to 77.46%, when the drying period increased from 1 to 4 h, respectively. For propane dryer, the accumulated weight loss of basil leaves significantly increased from 47.35 to 79.39%, when the drying period increased from 1 to 4.6 h, respectively. The results also indicate that the highest rate of weight loss was occurred at the first hour. It could be seen that the weight losses were 21.45, 52.19 and 47.35% for solar, diesel and propane dryers, respectively.

Regression analysis was aimed out to find a relation between the accumulated weight loss and types for different energy sources and the most appropriate forms were as follows:

11 $$ {\text{Diesel}}\,{\text{Dryer}}\,{\text{WL }} = { 8}.{44 } + { 48}.{95}\,{\text{ln}}\,{\text{T}}\,{\text{R}}^{{2}} = \, 0.{916} $$

12 $$ {\text{Propane}}\,{\text{Dryer}}\,{\text{WL }} = { 8}.{57 } + { 43}.{81}\,{\text{ln}}\,{\text{T}}\,{\text{R}}^{{2}} = \, 0.{937} $$

13 $$ {\text{Solar}}\,{\text{Dryer}}\,{\text{WL }} = \, 0.{49 } + { 28}.{34}\,{\text{ln}}\,{\text{T}}\,{\text{R}}^{{2}} = \, 0.{988} $$

### Moisture content

**Figure 8:**
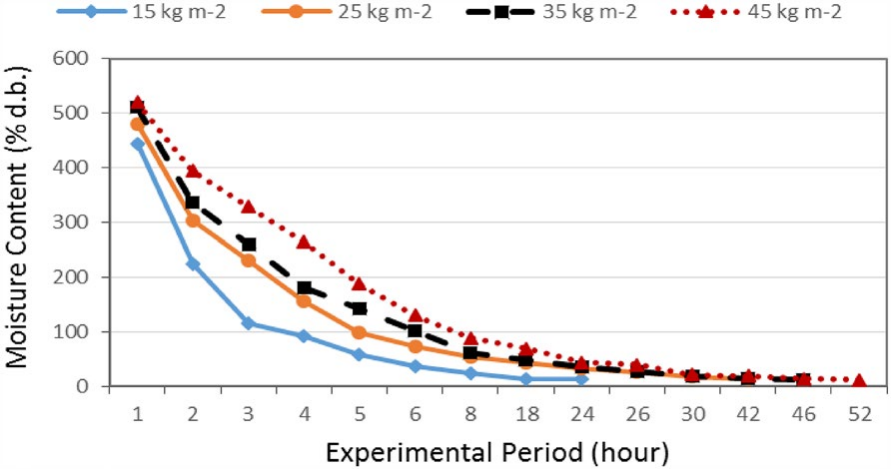
The moisture content of basil leaves for different loading rates during experimental period.

Figure 8 shows the moisture content of basil leaves that dried at different loading rates (15, 25, 35 and 45 kg m^−2^) during experimental period. The results indicate that the moisture content of basil leaves decreases with increasing drying period for all treatments. It could be seen that the moisture content of basil leaves significantly decreased from 443.84 to 12.67% d.b., when the drying period increased from 1 to 24 h at 15 kg m^−2^ loading rate. At 25 kg m^−2^ loading rate, the moisture content of basil leaves significantly decreased from 479.74 to 13.02% d.b., when the drying period increased from 1 to 42 h. At 35 kg m^−2^ loading rate, the moisture content of basil leaves significantly decreased from 510.73 to 13.55% d.b., when the drying period increased from 1 to 46 h. 45 kg m^−2^ loading rate, the moisture content of basil leaves significantly decreased from 519.87 to 11.97% d.b., when the drying period increased from 1 to 52 h. These results agreed with those obtained by^11,29,30^.

The drying temperatures affected both time of drying and final moisture content. The higher drying temperature with the shorter total drying time and the lower final moisture content of the dried basil leaves. This could be due to the vaporization of surface moisture from the material which became more efficient upon contact with more heat^31,32^.

The results indicate that the moisture content of basil leaves increases with increasing loading rate. It could be seen that, when the loading rate increased from 15 to 45 kg m^−2^, the moisture content of basil leaves significantly increased from 443.84 to 519.87% d.b., respectively, after 1 h operated for solar dryer. Also, it significantly increased from 12.67 to 43.68% d.b., when the loading rate increased from 15 to 45 kg m^−2^, respectively, after 24 h operated for solar dryer.

The results indicate that the highest rate of the decrease in moisture content of basil leaves (97.70%) was happened at the 45 kg m^−2^ loading rate. Meanwhile, the lowest rate of the decrease in moisture content of basil leaves (97.14%) was found at 15 kg m^−2^ loading rate. The trend of these results agreed with those obtained by^33^.

Multiple regression analysis was carried out to obtain a relationship between the moisture content as dependent variable and different both of loading rate and experimental period as independent variables. The best fit for this relationship is presented in the following equation:-

14 $$ MC = 170.31 + 3.56LR - 13.31T\quad {\text{R}}^{{2}} = 0.87 \, $$

where MC is the moisture content, %.

This equation could be applied in the range of 15 to 45 kg m^−2^ loading rate and from 1 to 24 h of experimental period.

### Drying rate

**Figure 9:**
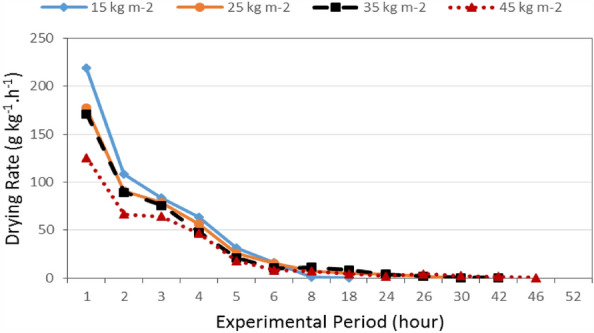
The drying rate of basil leaves for different loading rates during experimental period.

Figure 9 shows the drying rate of basil leaves that dried at different loading rates (15, 25, 35 and 45 kg m^−2^) during experimental period. The results indicate that the drying rate of basil leaves decreases with increasing drying period for all treatments. It could be seen that the drying rate of basil leaves significantly decreased from 219.54 to 0.24 g_water_ kg^−1^ h^−1^, when the drying period increased from 1 to 24 h at 15 kg m^−2^ loading rate. At 10 cm thickness basil layer, the drying rate of basil leaves significantly decreased from 177.36 to 0.43 g_water_ kg^−1^ h^−1^, when the drying period increased from 1 to 42 h. At 25 kg m^−2^ loading rate, the drying rate of basil leaves significantly decreased from 171.32 to 0.25 g_water_ kg^−1^ h^−1^, when the drying period increased from 1 to 46 h. At 35 kg m^−2^ loading rate, the drying rate of basil leaves significantly decreased from 125.31 to 0.31 g_water_ kg^−1^ h^−1^, when the drying period increased from 1 to 52 h. The results also show the relation between moisture content and drying rate for all treatments, the drying rate of basil leaves decreases with increasing moisture content. It could be seen that, when the moisture content increasing from 443.84 to 519.87% d.b., the drying rate significantly decreased from 219.54 to 125.31 g_water_ kg^−1^ h^−1^ at 15 and 45 kg m^−2^ loading rate, respectively, after 1 h of drying time. These results agreed with those obtained by^34^.

The results indicate that the highest rates of the decrease in drying rate of basil leaves were happened after 1 h of drying period. It could be seen that, the highest rates of the decrease in drying rate of basil leaves were 50.71, 48.82, 47.81 and 46.98% at 15, 25, 35 and 45 kg m^−2^ loading rate, respectively, after 1 h of drying period.

Multiple regression analysis was carried out to obtain a relationship between the drying rate as dependent variable and different both of loading rate and experimental period as independent variables. The best fit for this relationship is presented in the following equation:-

15 $$ DR = 113.88 - 0.71LR - 6.88T\quad {\text{R}}^{{2}} = 0.88 $$

where DR is the drying rate, g_water_ kg^−1^ h^−1^.

This equation could be applied in the range of 15 to 45 kg m^−2^ loading rate and from 1 to 24 h of experimental period.

### Quality of dried basil

**Figure 10:**
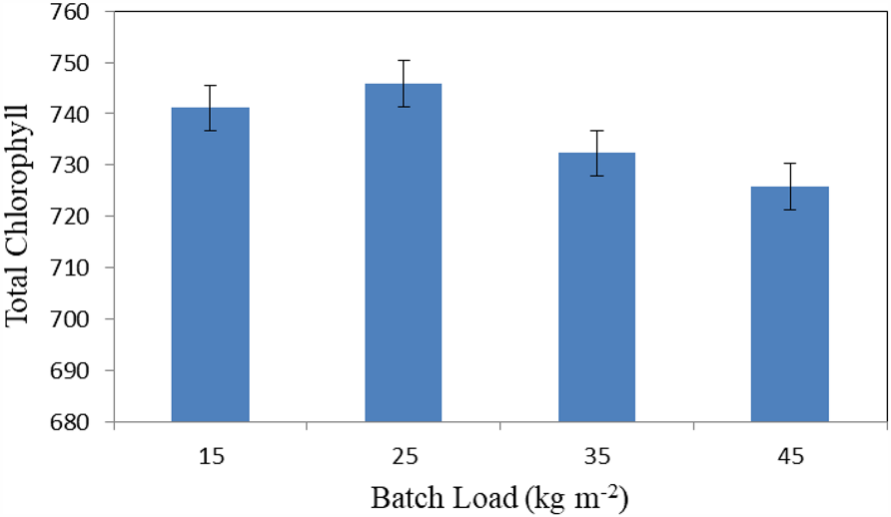
Total chlorophyll of basil leaves that dried at different batch loading rates.

Figure 10 shows the total chlorophyll of basil leaves that dried at different batch loading rates (15, 25, 35 and 45 kg m^−2^) at the end of experimental period. The results indicate that the total chlorophyll of basil leaves were 741.1, 745.9, 732.3 and 725.8 for 15, 25, 35 and 45 kg m^−2^ loading rate, respectively. The results also indicate that the highest value of total chlorophyll of basil leaves 745.9 was found of 25 kg m^−2^ batch loading rate. Meanwhile, the lowest value of total chlorophyll of basil leaves 725.8 was found of 45 kg m^−2^ batch loading rate.

**Figure 11:**
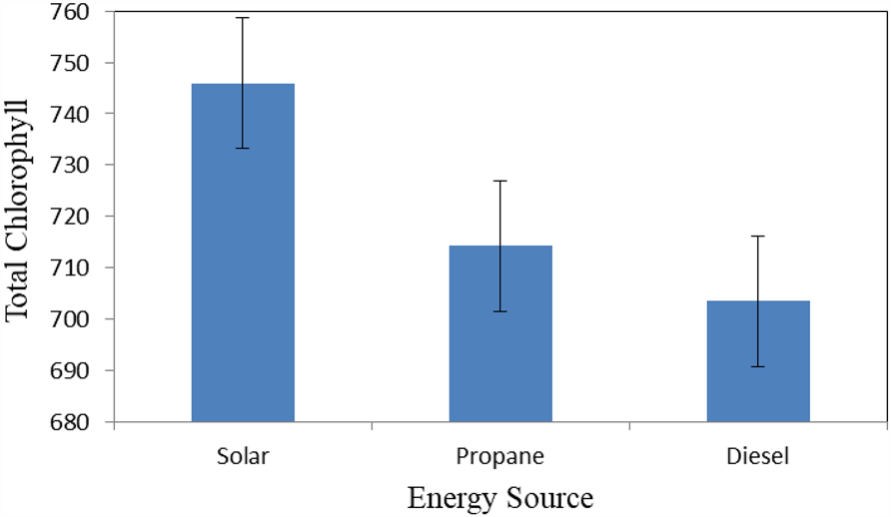
Total chlorophyll of basil leaves that dried under different energy sources.

Figure 11 shows the total chlorophyll of basil leaves that dried under different energy sources (solar, propane and diesel energies) at the end of drying period. The results indicate that the total chlorophyll of basil leaves were 745.9, 715.2 and 703.5 for solar, propane and diesel dryers, respectively. The results also indicate that the highest value of total chlorophyll of basil leaves 745.9 was found for solar dryer. Meanwhile, the lowest value of total chlorophyll of basil leaves 703.5 was found for diesel dryer.

**Figure 12:**
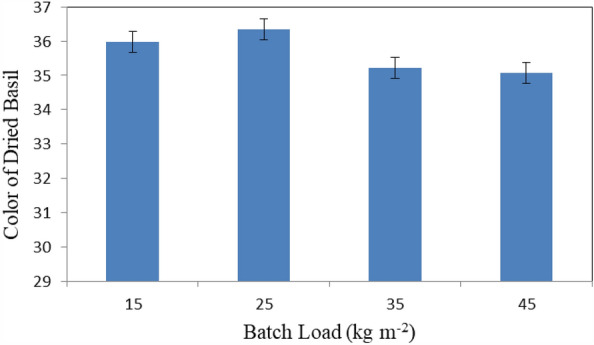
Color of basil leaves that dried at different batch loading rates.

Figure 12 shows the color of basil leaves that dried at different batch loading rates (15, 25, 35 and 45 kg m^−2^) at the end of experimental period. The results indicate that the color of basil leaves were 35.98, 36.35, 35.22 and 35.07 for 15, 25, 35 and 45 kg m^−2^ batch loading rate, respectively. The results also indicate that the highest value of color of basil leaves 36.35 was found of 25 kg m^−2^ batch loading rate. Meanwhile, the lowest value of color of basil leaves 35.07 was found of 45 kg m^−2^ batch loading rate.

**Figure 13:**
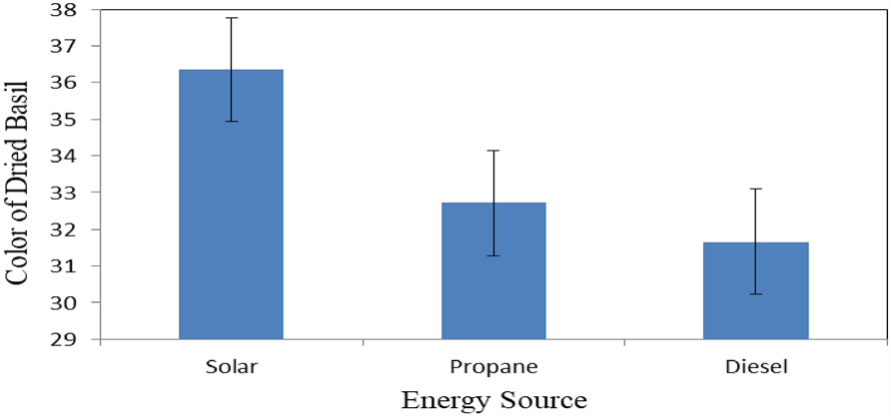
Color of basil leaves that dried under different energy sources.

Figure 13 shows the color of basil leaves that dried under different energy sources (solar, propane and diesel energies) at the end of drying period. The results indicate that the color of basil leaves were 36.35, 32.71 and 31.66 for solar, propane and diesel dryers, respectively. The results also indicate that the highest value of color of basil leaves 36.35 was found for solar dryer. Meanwhile, the lowest value of color of basil leaves 31.66 was found for diesel dryer.

### Basil essential oil content

**Figure 14:**
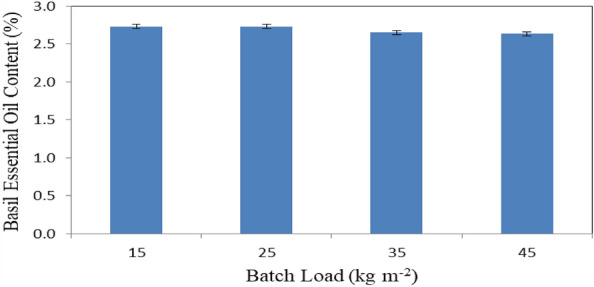
Basil essential oil content that dried at different batch loading rates.

Figure 14 shows the basil essential oil content that dried at different batch loading rates (15, 25, 35 and 45 kg m^−2^) at the end of experimental period, extracted by hydrodistillation. The results indicate that the basil essential oil content decreases with increasing batch loading rate. It could be seen that the basil essential oil content was non-significantly decreased from 2.73 to 2.63%, when the batch loading rate increased from 15 to 45 kg m^−2^. The results also indicate that the highest value of basil essential oil content 2.73% was found of 15 and 25 kg m^−2^ batch loading rate. Meanwhile, the lowest value of basil essential oil content 2.63% was found of 45 kg m^−2^ batch loading rate.

**Figure 15:**
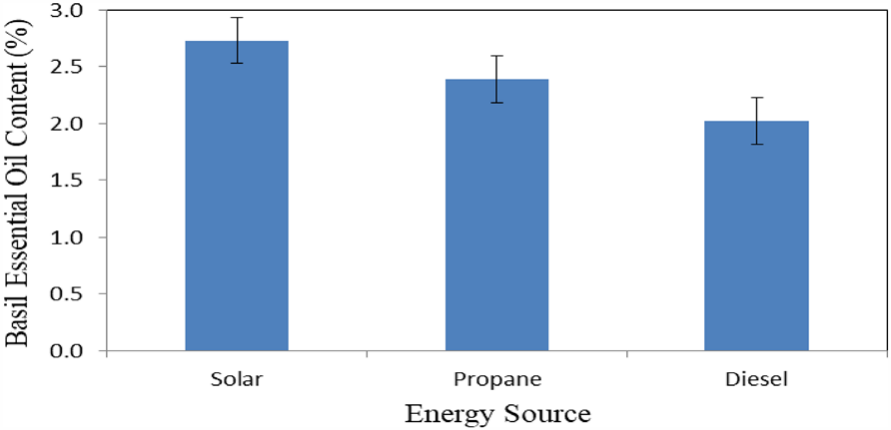
Basil essential oil content that dried under different energy sources.

Figure 15 shows the basil essential oil content that dried under different energy sources (solar, propane and diesel energies) at the end of drying period, extracted by hydrodistillation. The results indicate that the basil essential oil content were 2.73, 2.39 and 2.02% for solar, propane and diesel dryers, respectively. The results also indicate that the highest value of basil essential oil content 2.73% was found for solar dryer. Meanwhile, the lowest value of basil essential oil content 2.02% was found for diesel dryer. The highest essential oil yield obtained for solar dryer, the lowest essential oil yield occurred for diesel dryer. The trend of these results agreed with those obtained by^7^ whose mentioned that increasing the drying temperature significantly decreased the essential oil content.

Generally, according to^35–37^ essential oil yield of medicinal and aromatic plants is influenced by drying, whereas depending on the process time and temperature as well as the method, the oil yield could be increased and/or decreased. Furthermore, changes in the oil content are dependent on the plant species.

### Drying efficiency

**Figure 16:**
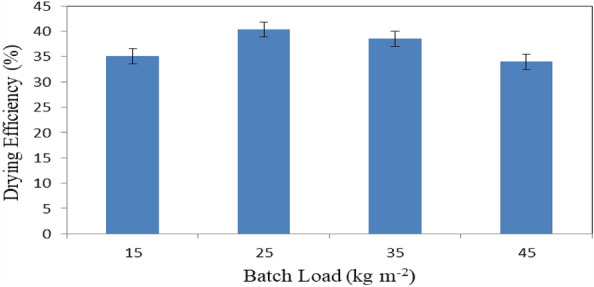
Drying efficiency of basil leaves at different batch loading rates.

Figure 16 shows the drying efficiency of basil leaves at different batch loading rates (15, 25, 35 and 45 kg m^−2^) at the end of experimental period. The results indicate that the drying efficiency of basil leaves were 35.06, 40.33, 38.52 and 33.98% for 15, 25, 35 and 45 kg m^−2^ loading rate, respectively. The results also indicate that the highest value of drying efficiency of basil leaves 40.33% was found of 25 kg m^−2^ batch loading rate. Meanwhile, the lowest value of total chlorophyll of basil leaves 33.98 was found of 45 kg m^−2^ batch loading rate.

**Figure 17:**
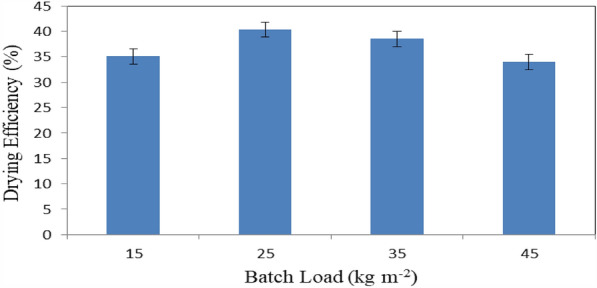
Drying efficiency of basil leaves under different energy sources.

Figure 17 shows the drying efficiency of basil leaves under different energy sources (solar, propane and diesel energies) at the end of drying period. The results indicate that the drying efficiency of basil leaves were 40.33, 46.15 and 51.07% for solar, propane and diesel dryers, respectively. The results also indicate that the highest value of the drying efficiency of basil leaves 51.07% was found for propane dryer. Meanwhile, the lowest value of the drying efficiency of basil leaves 40.33% was found for solar dryer. The trend of these results agreed with those obtained by^38^ whose found the highest drying efficiency obtained for gas dryer, the lowest the drying efficiency occurred for solar dryer. Also, these results agreed with those obtained by^39^.

### Volatile compounds of basil

**Figure 18:**
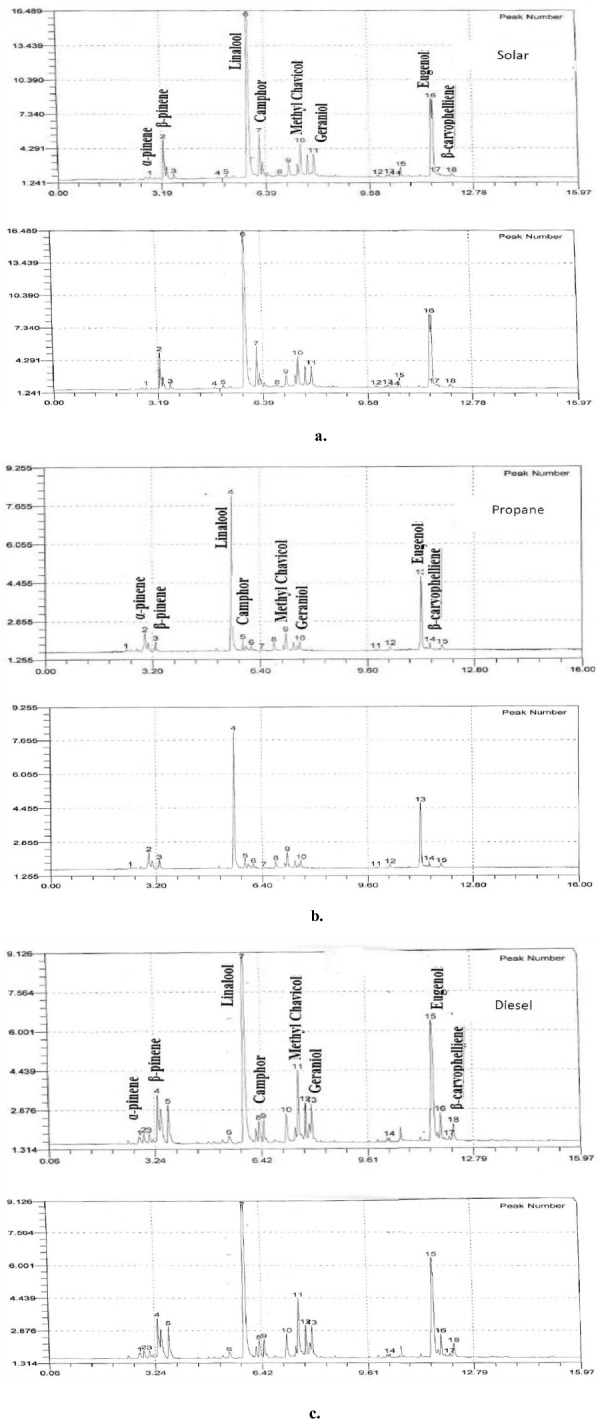
(**a**) A typical chromatogram of volatile composition of dry basil leaves in solar dryer. (**b**) A typical chromatogram of volatile composition of dry basil leaves in propane dryer. (**c**) A typical chromatogram of volatile composition of dry basil leaves in diesel dryer.

Table 3 shows the volatile compounds of basil that dried under different energy sources (solar, propane and diesel energies) at the end of drying period, extracted by hydrodistillation. A typical chromatogram of volatile composition of dry basil leaves is shown in Fig. 18a–c. The results indicate that the volatile losses which increased with higher temperature and lower drying time. The best results from a volatile composition point of view were obtained for sample convectively dried in solar dryer. It could be seen that the α-pinene and β-pinene were 0.62, 0.52 and 0.18 and 6.23, 8.10 and 3.23% for solar, propane and diesel dryers, respectively. The linalool, camphor and methyl chavicol were 30.85, 36.15 and 35.88, 4.74, 4.48 and 3.04 and 11.54, 8.36 and 7.08% for solar, propane and diesel dryers, respectively. The geraniol, eugenol and β-caryophelliene were 7.88, 5.14 and 8.68, 20.95, 19.14 and 18.40 and 2.99, 2.52 and 2.32% for solar, propane and diesel dryers, respectively. The results also indicate that the highest value of volatile compounds with found for solar dryer. The trend of these results agreed with those obtained by^40^ whose found that the volatile composition of basil leaves decreases with increasing the drying temperature.

**Table 3:** The volatile compounds of basil that dried under different energy sources.

Compound	Source of energy		
	Solar	Propane	Diesel
α-Pinene	0.62^b^	0.52^b^	0.18^a^
β-Pinene	6.23^b^	8.10^c^	3.94^a^
Linalool	39.85^b^	36.15^a^	35.88^a^
Camphor	4.74^b^	4.48^b^	3.04^a^
methyl chavicol	11.54^b^	8.36^a^	7.08^a^
Geraniol	7.88^b^	5.14^a^	8.68^c^
Eugenol	20.95^a^	19.14^a^	18.40^a^
β-Caryophelliene	2.99^a^	2.52^a^	2.32^a^

Means on the same row with different superscripts are significantly different (p < 0.05).

### Drying costs

Table 4 shows the fixed, variable, total costs and total costs per kg for the basil drying. It could be seen that the fixed costs were 5.77 LE h^−1^ for different dryer (solar, diesel and propane dryers). The variable costs were 13.96, 20.93 and 18.16 LE h^−1^ for solar, diesel and propane dryers, respectively. The total costs for basil drying were 19.73, 26.70 and 23.93 LE h ^- 1^ for solar, diesel and propane dryers, respectively. Also, the total costs per kg of basil drying were 8.77, 13.15 and 12.27 LE for solar, diesel and propane dryers, respectively. The total costs of basil dried in diesel dryer were 1.5 times higher than those basil dried in solar dryer, also the total costs of basil dried in propane dryer were 1.4 times higher than those basil dried in solar dryer. Besides the solar energy is a clean source of energy and environmental friendly used.

**Table 4:** The fixed, variable and total costs of basil drying using different sources of energy.

Item	Solar dryer				Diesel dryer	Propane dryer
	Loading rate (kg m^−2^)					
	15	25	35	45		
Fixed costs (L.E h^−1^)	5.77^a^	5.77^a^	5.77^a^	5.77^a^	5.77^a^	5.77^a^
Variable costs (L.E h^−1^)	13.96^a^	13.96^a^	13.96^a^	13.96^a^	20.93^c^	18.16^b^
Total costs (L.E h^−1^)	19.73^a^	19.73^a^	19.73^a^	19.73^a^	26.70^c^	23.93^b^
Total costs (L.E kg^−1^)	12.98^c^	8.77^b^	5.87^a^	4.78^a^	13.15^c^	12.27^c^

Means on the same row with different superscripts are significantly different (p < 0.05).

It could be noticed that the drying costs were affected by changing the batch load in the solar dryer, where it varied from 12.98 to 4.78 LE kg^−1^ when the batch load changed from 15 to 45 kg m^−2^, respectively.

## Conclusion

The experiment was carried out successively to investigate the effect of different loading rates on the energy required for basil drying with different sources of energy. It is concluded that the accumulated weight loss of basil leaves increased from 27.29 to 75.03, 21.45 to 75.56, 16.74 to 74.91 and 12.92 to 76.12% at 15, 25, 35 and 45 kg m^−2^ loading rate of basil, respectively. The highest rate of the decrease in moisture content of basil leaves (97.70%) was happened at the 45 kg m^−2^ loading rate of basil. The drying rate of basil leaves ranged from 125.31 to 219.54 g_water_ kg^−1^ h^−1^ for all treatments under study. The highest values of total chlorophyll and color of basil leaves 745.9 and 36.35 were found for solar dryer. The basil essential oil content was decreased from 2.73 to 2.63%, when the batch loading rate increased from 15 to 45 kg m^−2^. The highest value of the drying efficiency of basil leaves 51.07% was found for propane dryer. The total costs for basil drying were 19.73, 26.70 and 23.93 LE h^−1^ for solar, diesel and propane dryers, respectively. Also, the total costs per kg of basil drying were 8.77, 13.15 and 12.27 LE for solar, diesel and propane dryers, respectively. Besides the solar energy is a clean source of energy and environmental friendly used. The optimum conditions of basil drying rate, loading rate 25 kg m^−2^, 60 °C and 10% RH. Which gave higher efficiency and low energy requirements. Using propane saved 58.8% of than energy required from diesel fuel. Further studies should be carried out on the effect of a recirculation rate on drying efficiency. More studies could be on more different sources of heat. It is worthy to conclude that solar drying reduced the CO_2_ gas emission, where basil dried using fuel produced 0.5 kg CO_2_ for 1 kg dried and using propane fuel produced 0.04 kg CO_2_ for 1 kg dried product.

## Data Availability

The datasets used and/or analyzed during the current study available from the corresponding author on reasonable request.
